# Exploring a Cheese Ripening Process That Hinders Ochratoxin A Production by *Penicillium nordicum* and *Penicillium verrucosum*

**DOI:** 10.3390/biology13080582

**Published:** 2024-08-01

**Authors:** Alicia Rodríguez, Naresh Magan, Josué Delgado

**Affiliations:** 1 School of Agricultural Engineering, University of Extremadura, Avda. Adolfo Suárez s/n, 06007 Badajoz, Spain; 2 Instituto Universitario de Investigación de Recursos Agrarios (INURA), Universidad de Extremadura, Avda. de la Investigación s/n, Campus Universitario, 06006 Badajoz, Spain; 3 Applied Mycology Group, Environment and AgriFood Theme, Cranfield University, Cranfield MK43 0AL, Bedfordshire, UK; n.magan@cranfield.ac.uk; 4 Higiene y Seguridad Alimentaria, Instituto de Investigación de Carne y Productos Cárnicos (IProCar), Facultad de Veterinaria, Universidad de Extremadura, Avda. de las Ciencias s/n, 10003 Cáceres, Spain

**Keywords:** cheese, *Penicillium nordicum*, *Penicillium verrucosum*, ochratoxin A, technological parameters

## Abstract

**Simple Summary:**

Ochratoxin A (OTA), a nephrotoxic mycotoxin categorized as a possible carcinogenic agent, is a current problem in the cheese manufacturing industry. The main OTA producers are *Penicillium nordicum* and *Penicillium verrucosum*. Therefore, we aimed to investigate different cheese ripening phases to hinder OTA production. We elaborated cheese analogues, inoculated both fungal species, and performed two different ripening phases, at 10 and 15 °C. Despite the fact that both were able to grow, they did not express genes related to OTA production, and no OTA was detected during two weeks of ripening. Therefore, we show that, when the first phases of ripening are maintained at <15 °C, the possibility of OTA production is dramatically minimized. This information is of utmost importance for industries to enhance the safety of their cheese products, which positively impacts human health.

**Abstract:**

A lack of control of the technological abiotic parameters apparent during cheese manufacture, including temperature and relative humidity, results in this dairy product being prone to mold contamination. Sometimes, inoculant molds are used to obtain the characteristic sensory properties of this type of product. However, during the maturation process, some unwanted molds can colonize the ripening cheese and produce mycotoxins. Mycotoxigenic molds such as *Penicillium nordicum* and *Penicillium verrucosum* can colonize ripened cheeses, contaminating them with ochratoxin A (OTA), a nephrotoxic 2B toxin. Thus, the presence of OTA in cheeses could represent a hazard to consumers’ health. This study has evaluated the growth and OTA production of *P. nordicum* and *P. verrucosum* on a cheese analogue under simulated ripening conditions of 10 and 15 °C and 0.96 water activity (a*_w_*). Ecophysiological, molecular, and analytical tools assessed the mold growth, gene expression, and OTA production under these environmental conditions. Both species were able to effectively colonize the cheese under these ripening conditions. However, neither species expressed the *otapks* and *otanps* biosynthetic genes or produced phenotypic OTA. Therefore, these results suggest a relatively low risk of exposure to OTA for consumers of this type of cheese product. The conditions used were thus appropriate for cheese ripening to minimize the potential for contamination with such mycotoxins. An appropriate adjustment of the technological ripening parameters during such cheese manufacture could contribute to OTA-free cheeses.

## 1. Introduction

Many varieties of cheeses are produced worldwide, with a range of ripening and curing processes, depending on the region or country of origin. Each year, around nine million tons of cheese are consumed in the European Union. In 2021, the *per capita* cheese consumption in the EU was about 20 kg per person [1]. Fresh cheeses, such as feta or mozzarella, are consumed just after rennet coagulation without any maturation step, but most of them require a ripening stage for their manufacture. During ripening, cheese is susceptible to significant microbiological and biochemical changes that are influenced by several abiotic factors that have to be managed by the producer, especially temperature and relative humidity (RH). Usually, depending on the type of cheese processing, the temperature most often used is ≤15 °C [2,3,4,5,6].

The microbial population of cheeses during maturation mainly consists of lactic acid bacteria, yeasts, and filamentous molds. Focusing on filamentous molds, often, inoculants are added to improve the sensory characteristics of cheese during ripening, e.g., *Penicillium roqueforti* in blue-veined cheeses and *Penicillium camemberti* in Camembert cheeses. However, the effective control of the RH in the environment and the water activity (a*_w_*) of the cheese surface during the ripening is important in the manufacture of some types of cheeses to allow the beneficial molds to colonize the cheese surface. The control of these parameters is critical in ensuring that colonization by the beneficial molds occurs for the development of the appropriate cheese flavor and texture, and the avoidance of competition from spoilage molds linked to undesirable characteristics [7]. Amongst these spoilage molds, some of them are mycotoxigenic and often belong to the *Penicillium* genus, which is well adapted to growth on cheese matrixes [8].

The primary mycotoxin found in different cheeses is ochratoxin A (OTA). This mycotoxin has been detected in different commercial cheeses, reaching levels of up to 262.2 µg/kg [9,10]. Indeed, in some artisan Italian cave cheeses, >6000 µg/kg of OTA was found [11]. This is probably why cheeses are considered to be one of the three main food groups that can contribute to chronic dietary exposure to OTA [12]. It has also been shown that OTA can migrate from the rind to up to 1.6 cm into French semi-hard cheeses during production [13]. In addition, a recent scientific opinion released by the European Food Safety Authority (EFSA) has emphasized the necessity of more occurrence data on OTA in cheese paste in comparison to cheese rinds [12].

The milk used in these cheeses as a source of OTA is negligible as a consequence of the hydrolytic activity of rumen bacteria, which degrade a high percentage of OTA from feed [14]. However, fungal spores settling on the ripening cheeses are probably the main source of mycotoxigenic fungi that can contaminate the cheeses with OTA [15]. OTA can be produced in cheeses by contaminant species from *Penicillium* and *Aspergillus* genera, as recently reviewed by Kure and Skaar [15]. This mycotoxin has shown nephrotoxic, hepatotoxic, immunosuppressive, teratogenic, and carcinogenic effects on humans [16]. Because of these detrimental effects, OTA has been classified as a possible human carcinogen (group 2B) by the International Agency of Research on Cancer [17].

OTA biosynthesis requires two key genes: Non-Ribosomal Peptide Synthetase and Polyketide Synthase enzymes, coded by *otanps* and *otapks* genes, respectively [18], although other enzymes, such as halogenase, also play a key role. The gene expression always precedes the phenotypic production in filamentous fungi, therefore, the expression of these key genes can be used to predict whether OTA accumulation may occur in ripened cheeses, as has been demonstrated in dry-cured meat products [19]. It is thus of utmost interest to examine the ability of ochratoxigenic molds to colonize ripening cheeses and the potential for contamination with OTA. *Penicillium nordicum* and *Penicillium verrucosum* have been identified as species that have been isolated from the surface of ripening cheeses and can grow in protein-rich food [20,21]. Thus, the objectives of this work were to evaluate the effect of manufacture and the parameters related to the ripening of cheese on (a) lag phases prior to growth, (b) growth, (c) gene expression, and (d) OTA contamination of cheese analogues inoculated with *P. nordicum* or *P. verrucosum*. The information extracted will be of utmost interest to take corrective actions by manufacturers in the cheese industry.

## 2. Materials and Methods

### 2.1. Mold Strains

One strain of *P. nordicum* (FHSCC2) and one strain of *P. verrucosum* (FHSCC4) were used in this study. Both strains are in the Culture Collection of Food Hygiene and Safety at the University of Extremadura, (Cáceres) Spain. The strain *P. nordicum* (FHSCC2) and the strain *P. verrucosum* (FHSCC4) were isolated from the surface of dry-cured ham and they are OTA producers when incubated at 25 °C for 7 days on Malt Extract Agar, Potato Dextrose Agar, or Rose Bengal Chloramphenicol Agar, as well as on dry-cured ham and dry-fermented-sausage-based media [22,23]. The annual average temperature is 17.1 °C in Badajoz and 16 °C in Caceres; moreover, both provinces have a dry-summer subtropical climate (type CSA; Mediterranean climate) according to Köppen Climate classification [24].

### 2.2. Mold Inoculum Preparation

For inoculum preparation, Yeast Extract Sucrose agar (YES) was prepared by dissolving 20 g/L yeast extract, 150 g/L sucrose, 2 g/L MgSO_4_, and 20 g/L technical agar. Afterwards, the strains were inoculated on YES and incubated at 25 °C for 7 days. The spores were collected using 10 mL sterile water containing 0.05% Tween 80 (Acros Organics, Geel, Belgium) and rubbing the surface of the colonies with a sterile glass rod to remove conidia. The spore suspensions were kept in glycerol solutions at −80 °C and new starter cultures were used for each experiment.

The spore suspensions were counted using a hemocytometer (Fisher Scientific, Oxford, UK) and adjusted to 10^6^ conidia/mL for use as the inoculum.

### 2.3. Cheese Analogue Preparation and Experimental Conditions

Cheese analogues similar to semi-hard cheeses where molds grow on their surface were prepared for this study. These analogues were made as follows: For each analogue, 2 L of whole cow’s milk was mixed with 50 mL of distilled water containing 8 mL of citric acid (final concentration 1.25% (*w*/*v*)) and heated to 30 °C. This mixture was removed from the heat, and a starter culture, mainly consisting of yeasts, was added. Subsequently, 50 µL of liquid rennet (Home Stead. Farm Supplies, Banbury, UK) dissolved in 15 mL of distilled water was included in the mixture. This mixture was covered for 5 min. At this stage, the clots separated from the water were cut into pieces and heated again to 40 °C, taking off the mix from the heat for 5 min. The liquid was removed with the aid of a colander, and the curds were immersed in hot water (60 °C) in order to cook them until they appeared stretchy. The curds were removed from the water, and salt (2% NaCl) was added. The cheese was stretched out until it appeared smooth and skinny. To cool down the cheese, it was immersed in cold water at 10 °C. Ice was also added for 15 min to prevent the surface from becoming grainy.

**Figure 1:**
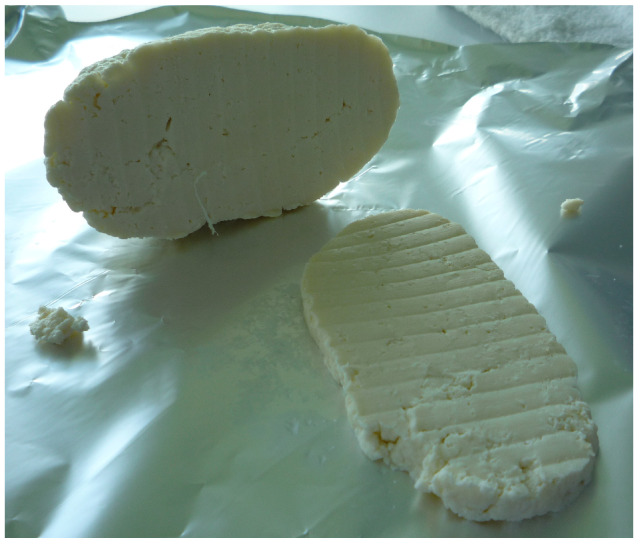
Cheeses made following the protocol described in Section 2.3 and slices resulting from cutting them.

After processing, the cooled cheeses were cut into approx. 4 mm thick slices and placed between squares of aluminum foil (Figure 1), previously sterilized. The slices were exposed to 254 nm UV light for 3–4 h to minimize any surface contamination that could have occurred during the analogue preparation. The sliced cheeses were then sprayed with isopropanol and kept in a laminar cabinet for 15 min to let them evaporate the isopropanol and were then placed into sterile 90 mm Petri plates and kept at 4 °C for 24 h in sterile sealed containers. The a*_w_* values of the cheese slices were checked using an Aqualab 3TE instrument (Decagon, Pullman, WA, USA). The pH of the cheese was also determined by using a pH meter BASIC 20 from Crison Instruments S.A. (Barcelona, Spain). The weight of the cheeses was *c.a.* 190 g with an a*_w_* value of 0.96 and pH between 5.2 and 5.5.

The cheese slices were centrally inoculated with 4 µL of the individual toxigenic mold inoculum (10^4^ spores per inoculation point). The slices were incubated at two different ripening temperatures, 10 and 15 °C, which are commonly used in the cheese ripening process [25]. The a*_w_* of these slices was kept constant at *c.a.* 0.96 throughout the incubation period through the use of glycerol and water dissolution, as previously described [26].

Destructive sampling was performed at different incubation times depending on the sample processing. The growth of the colonies was measured daily. For gene expression analysis, samples were taken after 7, 11, and 14 days of incubation. For OTA production, samples were collected after 7 and 14 days of incubation. All experiments were performed with three replicates per treatment and repeated twice.

### 2.4. Lag Phases Prior to Growth and Growth Assessment

The diameter of the growing colonies of all replicates and treatments was measured in two directions at a right angle to each other. These data were utilized for the determination of the lag phase prior to the growth (λ, days) and growth rate (µ, mm/day) of the strains. Primary modeling was carried out on the temporal colony radii data. In general, the data plots showed, after a lag phase, a linear trend with time. The mold colony radius was plotted against time, and linear regression was applied to obtain the growth rate as the slope of the line. The lag phase was calculated by equaling the regression line formula to the original inoculum size (diameter, mm) [22].

### 2.5. Gene Expression Studies

At each sampling time, the mycelium was harvested from the surface in a sterile flow bench, quickly frozen in liquid nitrogen, and stored at −80 °C until RNA extraction.

RNA isolation was essentially made according to the bead-beating method described by Rodríguez et al. [22]. To remove genomic DNA (gDNA) traces, the samples were treated with on-column DNase digestion using the RNase-Free DNase Set kit (Qiagen, Manchester, UK). The RNA concentration (ng/µL) and purity (A_260_/A_280_ ratio) were determined spectrophotometrically using a 2.5 µL aliquot on the Picodrop^TM^ (Spectra Services Inc., Ontario, NY, USA).

Once the RNA was extracted, reverse transcription (RT, cDNA synthesis) was performed using 5 μL of RNA (100 ng/µL), according to the manufacturer’s instructions from the Omniscript RT kit protocol (Qiagen, Hilden, Germany). Next, the resulting cDNA was stored at −20 °C until further use for real-time PCR (qPCR).

qPCR assays were used to amplify two key genes (*otapks* and *otanps*) of the OTA biosynthetic pathway as target genes, and *β-tubulin* was used as the control gene. The *otapks* and *β-tubulin* qPCR methods were previously optimized [22], while the qPCR based on the *otanps* gene was published previously [27]. SYBR Green^®^ dye (Takara Bio Inc., Shiga, Japan) was used. qPCR reactions were performed in a Rotor-Gene Q system (Qiagen, UK). Negative (sample without DNA) and positive (gDNA from the ochratoxigenic strains) controls were used in all runs. The reaction mixture was as follows: 6.25 μL of SYBR^®^ Premix Ex Taq^TM^ (Takara Bio Inc., Japan), 300 nM of each primer, and 2.5 μL of the cDNA template in a final volume of 12.5 μL. The thermal conditions included an initial denaturation step of 10 min at 95 °C and 40 cycles of 95 °C for 15 s and 60 °C for 30 s. After the last PCR cycle, a melting curve analysis for the PCR products was performed by heating to 72–95 °C and taking continuous measurement of the fluorescence to verify the PCR product. Quantification cycle (C_q_) determinations were automatically performed with the instrument using default parameters. The data from qPCR were analyzed using the software Rotor-Gene Q Series (2.1.0.9) Software (Qiagen, Hilden, Germany). Calculus of the relative expression of the *otapks* and *otanps* genes was carried out using the housekeeping gene *β-tubulin* as an endogenous control to normalize the quantification of the mRNA target to avoid errors caused by the multistage process required to extract, process, and detect mRNA. The expression ratio was calculated using the 2^−ΔΔCT^ method [28] after checking that the requirements to use it were met. This method allowed the calculation of the expression ratio of a target gene between a tested sample and its relative calibrator (control sample). In this study, the calibrator corresponded to samples incubated for 7 days.

### 2.6. Ochratoxin A Determination

OTA extraction was carried out according to the method reported by [29] with some modifications [9]. The cheese samples (2.5 g) were homogenized with 5 mL of acetonitrile after the addition of 20 µL of 18% sulfuric acid (*v*/*v*) to obtain a pH of 2.0 ± 0.5. The homogenized sample was then shaken horizontally for 5 min (200 rpm). Next, the samples were centrifuged at 5000 rpm for 3 min, and the supernatant was transferred into a new vial. Five mL of hexane was added, and the mixture was shaken horizontally at 200 rpm for 30 min. The hexane layer was removed, and this step was repeated twice with 5 mL of hexane. The organic phase was filtered through cellulose filters (0.45 µm pore size). The extract was evaporated to dryness at 60 °C and stored at −20 °C until analysis, whereupon 300 µL of mobile phase was added to dissolve the dry extract.

The OTA amounts produced by the two strains were quantified using HPLC. This equipment consisted of an Agilent 1200 series system (Agilent, Berkshire, UK) with a fluorescence detector (FLD, G1321A, Agilent, Santa Clara, CA, USA), an autosampler (ALS, G1329A, Agilent), autosampler thermostat (G1330B, Agilent), thermostated Column Compartment (G1316A, Agilent), on-line degasser (G1379B, Agilent), and a binary pump (G1312A, Agilent). The column was a Phenomonex^®^ Luna C_18_, 150 mm × 4.6 mm, 5 µm (Phenomenex, Macclesfield, UK) preceded by a pre-column (security guard, 4 mm × 3 mm cartridge, Phenomenex^®^ Luna, Hurdsfield Industrial Estate, Macclesfield, UK). The analysis was carried out in isocratic mode, and the mobile phase was water/acetonitrile/acetic acid (41:57:2 *v*/*v*/*v*). The flow rate was 1 mL/min and the injection volume was 50 µL. FLD detection was performed using 330 nm excitation and 460 nm emission wavelengths. The run time for the samples was 15 min, with OTA being detected at 5.75 min. The signals were processed by Agilent ChemStation software Ver. B Rev: 03.01 (317) (Agilent Technologies, Santa Clara, CA, USA). The limit of detection (LOD) was calculated using a 3:1 signal-to-noise ratio by taking the concentration of the analyte that produced a signal equal to the average background (Sblank) plus three times the standard deviation (sblank) of the blank: LOD = Sblank + 3sblank, whilst the limit of quantification (LOQ) was calculated as LOQ = Sblank + 10sblank. The LOQ and LOD of the method were 1.5 and 0.5 ppb, respectively [NO_PRINTED_FORM] [30].

### 2.7. Statistical Analysis

All data were analyzed using IBM SPSS for Windows v.15.0 software (IBM Corporation, Armonk, NY, USA). The data normality test was performed by using the Shapiro–Wilk test (*n* < 50). None of the data followed a normal distribution. The non-parametric data analysis was performed using the Kruskal–Wallis rank sum test. The Mann–Whitney U test was then applied to compare the median values obtained. The statistical significance was set at *p* < 0.05.

## 3. Results and Discussion

**Figure 2:**
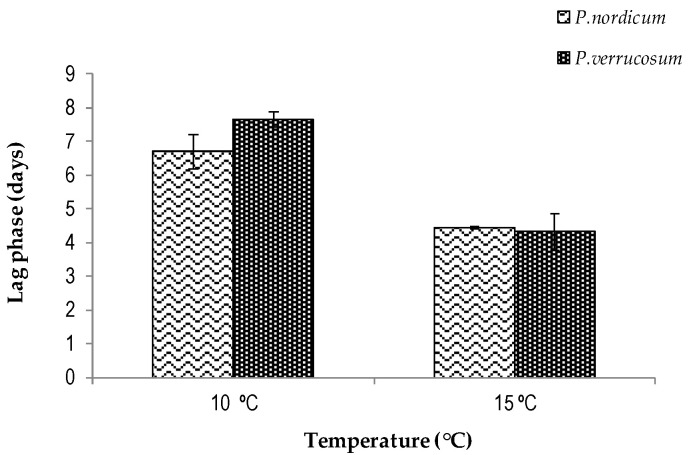
Comparison of lag phases prior to growth of *Penicillium nordicum* (FHSCC2) and *Penicillium verrucosum* (FHSCC4) on cheese analogues at 10 and 15 °C over a 14-day incubation period on ripening cheese analogues.

Figure 2 compares the lag phases prior to growth for the two species examined. Overall, they were shorter at 15 °C than at 10 °C (*p* ≤ 0.05) for both species. However, there were no significant differences at each steady-state temperature. There are no previous data on the lag phases for these two species on cheese analogues.

The cheese analogues contribute to simplifying ecophysiological studies. Although these analogues contain the same wild microbiological population as industrial cheeses, the biochemical reactions are simpler. In our study, the shorter ripening and the use of cold ripening make the results extracted only apply to cheeses that have undergone similar ripening, not being applicable to those varieties that require higher temperatures at the stated a*_w_* tested.

Although no data have been reported for cheese analogues, there are some data from studies on the colonization of cured meats [23]. They have reported differences in the lag phases between these two species when colonizing cured meats, with *P. nordicum* having shorter lag times than *P. verrucosum*. In addition, they found that at 0.97 a*_w_* *P. nordicum* FHSCC2 had slightly different lag times at 10 and 15 °C. *P. verrucosum* FHSCC4 was more sensitive and had significant differences in lag phases between these two temperatures. This may be partially due to the differences in the nutritional matrices or a better adaptation of the two mold strains to the dry ham that was the source of isolation.

However, it should be taken into account that the ripening of the analogous cheese takes place between 10 and 15 °C, and the two strains produced OTA at 25 °C for 7 days on laboratory culture media [23]. Temperature differences can significantly influence the growth of the fungal population and the production of OTA in the case of artisanal cheese maturation stages and outside the cold storage chain.

**Figure 3:**
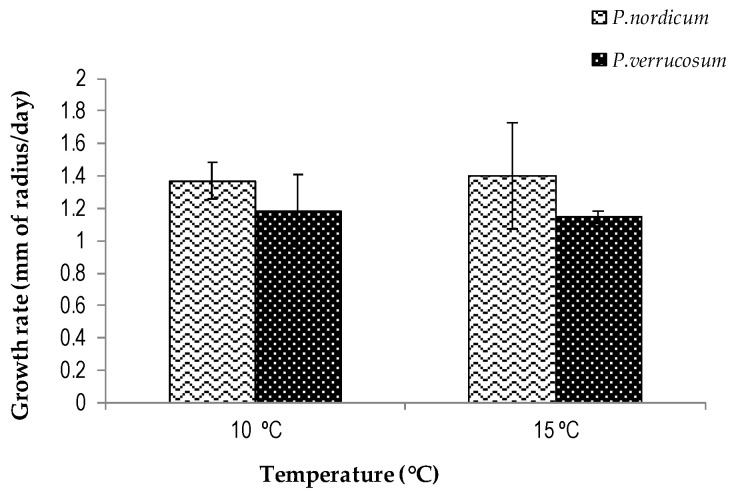
Growth rates of *Penicillium nordicum* (FHSCC2) and *Penicillium verrucosum* (FHSCC4) on cheese analogues (10 and 15 °C) over a 14-day incubation period simulating the cheese ripening.

The colonization of the cheese analogues at 10 and 15 °C by the two Penicillium species is shown in Figure 3. Both species were able to effectively colonize the cheese surface at both temperatures. The growth rate of the *P. nordicum* strain was slightly faster than that of the *P. verrucosum* strain. However, there were no statistically significant differences between them (*p* > 0.05). Additionally, there was no effect of ripening temperature (10, 15 °C) on colonization. In contrast, on dry-cured sausage at 0.94 a*_w_*, the strain of *P. verrucosum* had a faster colonization rate than *P. nordicum* at 15 °C [23]. Similar differences between these two fungal species were also found by Leggieri et al. (2020) in Grana cheese [31]. They found that the optimum growth conditions were at 15 and 20 °C, which were much more favorable than 10 °C. However, the a*_w_* values of the Grana cheese slices were 0.93 a*_w_*, lower than the 0.96 a*_w_* in the cheese analogues in the present study. Both of these Penicillium species are classified as xerotolerant or xerophilic. However, we know that *P. nordicum* grows much better in high-salt environments than *P. verrucosum*. The former species was thus found to be very resilient and adapted to the colonization of cured meat products [32]. The differences may also concern the differences in the cheese and dry-cured sausage matrices. Therefore, ecological, nutritional, and physicochemical parameters may influence the colonization of these matrices.

Although the microbial diversity of artisanal cheeses has been ever more extensively explored over recent years, to the best of our knowledge, most of the efforts regarding OTA production in cheeses have been made on OTA contamination surveys [9,10,33,34,35], instead of assessing the ability of ochratoxigenic species to contaminate cheese matrices. In fact, the information about the growth of ochratoxigenic Penicillium species and production of this mycotoxin throughout the ripening process related to different combinations of a*_w_* and temperature is limited and has not been performed on cheese matrixes [36,37]. Recent studies have focused on the ability of various molds isolated from cheeses to grow and produce OTA on non-cheese matrixes, and surveys of OTA in different artisanal cheeses at different maturation stages and temperatures have also been performed [13,31,35,38]. These studies are of utmost interest to better comprehend the molds’ ability to produce OTA, as well as the levels of contamination in cheese, since the presence of this mycotoxin in cheeses may pose an important health risk [11,12,39]. For instance, Rodríguez-Cañás et al. (2023) [34] found OTA in 3 varieties of cheeses of the 4 tested at concentrations that may pose a risk to human health and Anelli et al. (2019) [11] detected OTA in the rind of 6 of out 22 of tested cheese samples. Recently, methods for the detection of filamentous fungi in artisanal cheeses have been reviewed [39]. In this review, it has been highlighted that the abundance of artisanal cheeses influences the composition and fungi population on the cheese surface, which in turn can significantly contribute to desirable sensory qualities, while also contributing to defects, particularly during ripening, and risks associated with the production of mycotoxins. Nevertheless, it is worth mentioning that no available studies focus on the potential influence of the first stages of cheese ripening on the ability of *P. nordicum* and *P. verrucosum* to grow and produce OTA. Additionally, the present study has assessed the *otanps* and *otapks* gene expression to evaluate and predict OTA production under these environmental conditions. It deserves to be highlighted that, in this work, the whole process of cheese making has been performed similarly to the methods followed at the cheese factories, including rennet and all of the required ingredients, as described in Section 2.3. This fact allows for the extraction of reliable and transferable information, to fulfill the EFSA opinion requirements [12], and to be used by the cheese industry to prevent OTA production by these two ochratoxigenic species.

In regard to *P. nordicum* and *P. verrucosum* optimal growth conditions, a recent study has shown that they were 20 °C and 0.99 a*_w_* on Czapek Yeast Agar (CYA) [36]. These varied when both mold species grew on Malt Extract Agar at the same a*_w_* level, being at the optimum temperature of 25 °C [37]. This makes sense, since as it has been previously reported the nutritional composition affects fungal growth enormously [40]. In this work, the effect of both temperature and a*_w_* conditions related to the first stages of the ripening cheese process [2,3,4,5,6] on growth, gene expression, and OTA production of *P. nordicum* and *P. verrucosum* has been studied. Knowledge about the ecophysiology of both species at the first stages of cheese processing is critical to implementing preventive actions before OTA becomes present at dangerous levels.

The quantification of relative gene expression of the strain of both species showed no *otapks* or *otanps* gene expression at any of the three sampling times when colonizing the cheese analogues (7, 11, and 14 days). The use of a housekeeping gene as an endogenous control (*β-tubulin*) provides a means of avoiding any false-negative results. The quantification of these biosynthetic genes for these two molds has not previously been carried out in cheese. Previous studies with *P. nordicum* FHSCC2 colonizing cured meat substrates displayed stimulation of the expression of these two genes when colonizing dry-cured-ham-based media with a similar a*_w_* value, but at 25 °C [22]. Therefore, ecological factors, as well as the nutritional components of the medium, dramatically affect gene expression [41,42].

Therefore, as expected, the quantification of OTA production by the strains of these two mold species showed no contamination at the two incubation temperatures examined (Table 1). This parallels the lack of expression of key regulatory genes involved in OTA biosynthesis, regardless of the sampling time. Previously, Leggieri et al. [36] found no OTA production by *P. verrucosum,* even at 20 °C, at a similar a*_w_* level in CYA. However, *P. nordicum* was able to produce small quantities of OTA in two a*_w_* conditions (0.99 and 0.96 a*_w_*) and 15 °C and 20 °C, respectively, in the same culture medium [36]. The different conditions used in this previous study, especially the use of a defined artificial medium, may have resulted in the effect on OTA production. More useful are the data that have been obtained in cheese matrices, such as Grana or Comté cheeses. In Grana cheese at lower a*_w_* levels (0.93 a*_w_*) than those reported in the present study, *P. nordicum* was not able to produce OTA at either 10 or 15 °C during a 21-day incubation period [31], which is similar to that found in the present study. Interestingly, in that study, the maximum OTA production was detected at 25 °C, despite displaying a lower growth rate than that reported at 15 °C. However, under these conditions, *P. verrucosum* was able to produce OTA at 15 °C but not at 10 °C [31]. However, this mold strain produced much lower quantities of OTA at 15 °C than at 20 °C, despite the fact that this species grew similarly at 15 and 20 °C. In another type of matured cheese, Comté cheese (0.95 a*_w_*), *P. verrucosum* was able to produce OTA after 28 and 7 days at 8 and 20 °C, respectively [13].

The cheese analogue made in the present study contained 2% NaCl. Grana cheese usually contains about 1.4–1.8% NaCl [43], whilst Comté cheese must contain at least 0.6% NaCl [44]. The NaCl concentration has been reported as an OTA production promoter. In fact, the maintenance of Cl^−^ homeostasis seems to be an ecological reason for the biosynthesis of OTA [32]. Therefore, slight physicochemical differences among these matrices could be responsible for the impacts on OTA production. Additionally, the intra-species differences seemed to be crucial for the different behaviors observed with regard to OTA production. It appears to be very important that the processing of such cheeses is performed at ≤15 °C and 0.96 a*_w_* to ensure that the chances of any contamination with OTA can be prevented, even if these penicillia are able to colonize the ripening.

## 4. Conclusions

In the case of artisanal cheese maturation stages and outside the cold chain of storage, *P. nordicum* and *P. verrucosum* can produce the mycotoxin ochratoxin A, which represents a risk to the health of the consumers (nephrotoxic 2B toxin); moreover, this situation could be favored by the dry-summer subtropical climate in the Badajoz and Cáceres provinces of Spain. This study contributes to our knowledge about the ochratoxigenic *P. nordicum* and *P. verrucosum* behavior during semi-hard cheese ripening. Both species were able to effectively colonize the cheese under the ripening conditions. However, neither species expressed the *otapks* and *otanps* biosynthetic genes or produced phenotypic OTA. These results suggest a relatively low risk of exposure to OTA for consumers of this type of cheese product. It seems to be very important that the processing of such cheeses is performed at ≤15 °C and 0.96 a*_w_* to ensure that the chances of any contamination with OTA can be prevented, even if these penicillia are able to colonize the ripening surface. Additionally, this work points to a timeframe of two weeks to take corrective action with no risk of OTA production in cheese when ripening at the described conditions. These findings could help us to adjust the technological ripening parameters during cheese manufacture to elaborate safe cheese products.

## Figures and Tables

**Table 1:** Ochratoxin A production by *Penicillium nordicum* (FHSCC2) and *Penicillium verrucosum* (FHSCC4) in analog cheese at 7 and 14 days of ripening, at 10 and 15 °C, detected by HPLC-Fluorescence. (LOD: Limit of detection = 0.5 ppb).

Mold Species	OTA Concentration			
	7 Days		14 Days	
	10 °C	15 °C	10 °C	15 °C
*Penicillium nordicum*	<LOD			
*Penicillium verrucosum*	<LOD			

## Data Availability

Data available upon request from the authors.
